# Misperceiving Bullshit as Profound Is Associated with Favorable Views of Cruz, Rubio, Trump and Conservatism

**DOI:** 10.1371/journal.pone.0153419

**Published:** 2016-04-29

**Authors:** Stefan Pfattheicher, Simon Schindler

**Affiliations:** 1 Ulm University, Ulm, Germany; 2 Kassel University, Kassel, Germany; University of Georgia, UNITED STATES

## Abstract

The present research investigates the associations between holding favorable views of potential Democratic or Republican candidates for the US presidency 2016 and seeing profoundness in bullshit statements. In this contribution, bullshit is used as a technical term which is defined as communicative expression that lacks content, logic, or truth from the perspective of natural science. We used the Bullshit Receptivity scale (BSR) to measure seeing profoundness in bullshit statements. The BSR scale contains statements that have a correct syntactic structure and seem to be sound and meaningful on first reading but are actually vacuous. Participants (*N* = 196; obtained via Amazon Mechanical Turk) rated the profoundness of bullshit statements (using the BSR) and provided favorability ratings of three Democratic (Hillary Clinton, Martin O’Malley, and Bernie Sanders) and three Republican candidates for US president (Ted Cruz, Marco Rubio, and Donald Trump). Participants also completed a measure of political liberalism/conservatism. Results revealed that favorable views of all three Republican candidates were positively related to judging bullshit statements as profound. The smallest correlation was found for Donald Trump. Although we observe a positive association between bullshit and support for the three Democrat candidates, this relationship is both substantively small and statistically insignificant. The general measure of political liberalism/conservatism was also related to judging bullshit statements as profound in that individuals who were more politically conservative had a higher tendency to see profoundness in bullshit statements. Of note, these results were not due to a general tendency among conservatives to see profoundness in everything: Favorable views of Republican candidates and conservatism were not significantly related to profoundness ratings of mundane statements. In contrast, this was the case for Hillary Clinton and Martin O’Malley. Overall, small-to-medium sized correlations were found, indicating that far from all conservatives see profoundness in bullshit statements.

## Introduction

Bullshit is prevalent in all our lives. Yet, individuals differ in their sensitivity to bullshit, or in the way they see profoundness in bullshit statements [1]. The present research examines the relations between judging bullshit statements as profound and individuals’ favorable views of potential Democratic or Republican candidates for the US presidency 2016, as well as their general political liberal/conservative attitude.

Various forms of bullshit exist [2]. The reader might think of sincere or insincere exaggerations, nonsense statements, or blatant lies. In this work we focus on a specific form of bullshit, namely, pseudo-profound bullshit. In doing so, we build on the seminal work of Pennycook and colleagues [1] who provide a detailed conceptual and empirical analysis of pseudo-profound bullshit. Consider the following sentence, which appears to be sound and have a deep meaning on first reading but is actually vacuous: “Hidden meaning transforms unparalleled abstract beauty.” (p. 549, [1]) As shown in this example, pseudo-profound bullshit statements have a correct syntactic structure and are not trivial. However, they lack content, logic, or truth from the perspective of natural science (for real world examples, see [1]). As such, and in line with Pennycook and colleagues [1], bullshit is used as a technical term, defined as communicative expression that lacks plausibility and truth. Of note, we do not examine bullshit in a colloquial sense, such as when blatant lies or exaggerated stories are told. The present work focuses on pseudo-profound bullshit statements, that is, communicative expressions that appear to be sound and have a deep meaning on first reading but actually lack plausibility and truth from the perspective of natural science.

In general, rather than focusing on who talks bullshit, the present research takes a look at who considers pseudo-profound bullshit as profound. We predict that conservatives (in contrast to liberals) have a higher tendency for pseudo-profound bullshit receptivity. This prediction is based on the following two assumptions: First, given that pseudo-profound bullshit statements are not easy to read and to understand, individuals need the ability to detect that bullshit statements are ultimately meaningless and lack truth [1]. As Pennycook and colleagues demonstrated, the ability increases the more individuals use reflective and critical thinking [1]. Congruently, accepting information as true rather than false increases when the intuitive, automatic thinking mode is stimulated [3,4]. In order to detect pseudo-profound bullshit, however, individuals need to process statements using a reflective and critical thinking mode [1]. Second, research has shown that conservative attitudes are related to relying on intuitive thinking styles [5] while cognitive complexity (i.e., the tendency to construct a variety of perspectives for viewing an issue) is avoided [6,7]. These findings correspond to results showing a negative relation between conservatism and need for cognition [8] and cognitive ability [9].

Thus, based on the assumptions that individuals need to process pseudo-profound bullshit statements in a reflective and critical thinking mode to detect their vacuous content whereas conservatives compared to liberals are less likely to engage in the reflective and critical thinking mode but are more likely to use the (in this case maladaptive) intuitive thinking mode, we expect that political conservatism is related to judging bullshit statements as profound. Congruently, we expect that the more individuals have favorable views of persons talked about as potential Republican (conservative) candidates for US president the more they see profoundness in bullshit statements. These assumptions are tested in the study reported below. To exclude the possibility that conservatives are more likely to see profoundness in statements in general, we additionally include simple, mundane statements in our study. That is, conservatism should not be significantly related to seeing profoundness in mundane statements.

## Materials and Method

### Participants

We obtained complete data from 196 US-American individuals (43.4% women; *M*_age_ = 36.4) who participated in an online study via Amazon Mechanical Turk, a service where researchers can post jobs (such as responding to a questionnaire) which can be completed by users of Amazon Mechanical Turk (cf. [10]). One participant began the survey but did not complete it; results did not change when this individual was excluded from the analyses. No participant was removed from the reported analyses. Only demographic information about sex, age, and in what country participants live was collected. The data set can be found in S1 File.

In line with [11] the study was conducted in full accordance with the Ethical Guidelines of the German Association of Psychologists (DGPs) and the American Psychological Association (APA). Moreover, by the time the data were acquired in January of 2016 it was also not customary at Ulm University, Kassel University, nor at most other German universities to seek ethics approval for simple studies on personality and attitudes. The study exclusively makes use of anonymous questionnaires. No identifying information was obtained from participants. The participants were explicitly informed that the data are treated confidentially. Every participant had to agree to the following statements: "I understand that my participation is voluntary and that I may withdraw from the study at any time without explanation;" and "I hereby confirm that I am at least 18 years old, and that I agree to take part in this study." Moreover, it was possible to easily withdraw from the study at any time by closing the internet browser.

### Pseudo-profound bullshit

The Bullshit Receptivity scale (BSR) by Pennycook and colleagues [1] was used to assess pseudo-profound bullshit receptivity. The BSR includes 10 sentences that have a correct syntactic structure and seem to be profound and meaningful on first reading but are actually vacuous. Participants were asked how profound (in terms of deep meaning and of great and broadly inclusive significance) they consider each sentence to be (cf. [1]). A sample item reads: “Imagination is inside exponential space time events.” Open access to all items is provided by Pennycook and colleagues (see [1]). Participants rated the profoundness of each statement on a 5-point Likert-scale ranging from 1 = not at all profound, through 2 = somewhat profound, 3 = fairly profound, 4 = definitely profound, to 5 = very profound. The BSR had good reliability (Cronbach’s α = .87), a mean of 2.66, and an adequate standard deviation of 0.85.

### Simple mundane statements

These statements were assessed using the items provided by Pennycook and colleagues (see [1]). Participants were again asked how profound they considered each sentence. A sample item reads: “A wet person does not fear the rain.” Participants rated profoundness on the same Likert-scale as was used for the BSR. The mundane statements scale had good reliability (Cronbach’s α = .87), a mean of 3.16, and an adequate standard deviation of 0.83. The Pearson correlation of the BSR and mundane statements was .52 (*p* < .001), suggesting an underlying factor reflecting seeing profoundness in something.

### Favorability ratings of candidates

Participants were asked to rate the favorableness of three potential Democratic candidates and three Republican candidates for US president. The selection of the candidates was based on election possibility, that is, the three candidates from each party who polled highest nationwide (as of January 20, 2016) based on the HuffPost Pollster database were selected. For the Democratic Party these were Hillary Clinton, Martin O’Malley, and Bernie Sanders. For the Republican Party these were Ted Cruz, Marco Rubio, and Donald Trump.

To obtain the favorability ratings of the six candidates, participants read: “Next, you will look at the names of people discussed as potential Democratic [Republican] candidates for US president.” Whether a participant started with the Democratic or Republican candidates was determined at random. Participants rated the candidates on a 5-point Likert scale ranging from 1 = very unfavorable, through 2 = somewhat unfavorable, 3 = neither unfavorable nor favorable, 4 = somewhat favorable, to 5 = very favorable. If participants had not heard of a particular candidate or did not have an opinion they were asked to not mark anything. Means and standard deviations were as follows: Hillary Clinton (*M* = 2.76, *SD* = 1.43), Martin O’Malley (*M* = 2.54, *SD* = 1.03), Bernie Sanders (*M* = 3.53, *SD* = 1.47), Ted Cruz (*M* = 2.13, *SD* = 1.29), Marco Rubio (*M* = 2.42, *SD* = 1.24), Donald Trump (*M* = 1.94, *SD* = 1.39).

### Political liberalism/conservatism

The commonly used single item of “Where would you put yourself on a continuum from liberal to conservative?” was used to assess political liberalism/conservatism. A 7-point Likert scale was used. The scale end-points read (1) liberal and (7) conservative (*M* = 3.33, *SD* = 1.83).

## Results

Given that some of the variables were right-skewed (e.g., the majority of participants had rather an unfavourable view of Donald Trump) Spearman’s rho was used to produce nonparametric correlations.

**Fig 1:**
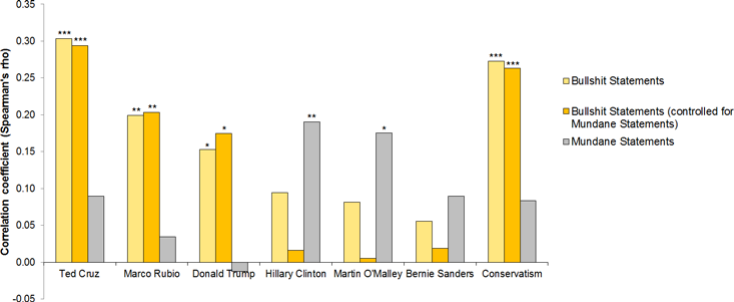
Spearman’s rho correlations among favorability ratings of the six candidates, conservatism, and seeing profoundness in bullshit and mundane statements. Note: * *p* < .05, ** *p* < .01, *** *p* < .001 (two-tailed).

As shown in Fig 1, favorable views of Ted Cruz, Marco Rubio, and Donald Trump were positively related to judging bullshit statements as profound. The strongest correlation was found for Ted Cruz. No significant relations were observed for the three Democratic candidates (Hillary Clinton, Martin O’Malley, and Bernie Sanders). The general measure of political conservatism was also positively related to judging bullshit statements as profound. Of note, all relations held in terms of associations and significance levels when mundane statements were controlled (see Fig 1).

In contrast, favorable views of Marco Rubio, Ted Cruz, and Donald Trump were not significantly related to judging mundane statements as profound. This also holds for Bernie Sanders. The picture was different for Hillary Clinton and Martin O’Malley. The more favorable views participants had of Hillary Clinton and Martin O’Malley, the more they saw profoundness in mundane statements.

## Discussion

Individuals differ in their tendency to see profoundness in bullshit [1]. The present research examined who is likely to judge pseudo-profound bullshit as profound by focusing on political attitudes and favorability ratings of potential Democratic and Republican candidates for US president. We considered it possible that conservatives are more receptive to pseudo-profound bullshit than liberals. In fact, the results revealed that holding favorable views of three potential Republican candidates for US president (Ted Cruz, Marco Rubio, and Donald Trump) was positively related to judging bullshit statements as profound. In contrast, non-significant relations were observed for the three Democratic candidates (Hillary Clinton, Martin O’Malley, and Bernie Sanders). A general measure of political liberalism/conservatism was also related to judging bullshit statements as profound. Specifically, the more individuals considered themselves to be politically conservative, the stronger their tendency to see profoundness in the bullshit statements presented. Of note, these results were not due to a general tendency among conservatives to see profoundness in everything: Favorable views of Republican candidates and conservatism were not significantly related to profoundness ratings of mundane statements. However, this was the case for two Democratic candidates: Favorable views of Hillary Clinton and Martin O’Malley were positively related to seeing profoundness in mundane sentences.

Recently, Pennycook and colleagues established a conceptual and empirical basis for the study of pseudo-profound bullshit [1]. However, research on bullshit is still in its infancy, likely because little time has passed since this seminal paper was published but certainly not because of its insignificance. Indeed, bullshit seems to be prevalent in all our lives [2], so it is reasonable to argue for scientific investigation into bullshit. As such, and given that little is known about who sees profoundness in bullshit, the present research extends knowledge in this field of study.

In the following paragraphs, we emphasize what can and what *cannot* be deduced from the study. First of all, we found small-to-medium sized correlations between holding favorable views of Republican candidates, conservatism, and bullshit receptivity. Given these effect sizes, it must be noted that far from all conservatives see profoundness in pseudo-profound bullshit statements. Certainly, some liberals also see profoundness in bullshit statements, while some conservatives may clearly reject profoundness in bullshit statements. Nevertheless, there is an overall tendency for conservatives relative to liberals to see profoundness in bullshit statements.

Second, we want to note that the sample of the present study probably is not representative of the US as our study is restricted to the specific sample of Amazon Mechanical Turk workers and has a relatively small sample size for an online survey. Thus, one cannot make inferences about the entire population of the US (or other populations of other countries). However, this does not undermine the significance of the present research. As is usual in psychological studies, associations between constructs are tested. Whether these associations hold for different populations is surely interesting, but beyond the scope of most psychological studies, including the present research. In this context, we also want to note that some participants might be non-naïve or trustworthy, as some participants regularly and systematically participate in online studies via the Amazon Mechanical Turk platform [12]. Nonetheless, research shows that valid results in psychological research can be obtained using Amazon Mechanical Turk [10,13].

Third, we want to emphasize that the present study is correlational in nature. Thus, no causal inferences can be drawn. One cannot conclude that conservatism leads to bullshit receptivity. What can be concluded is that conservatism is positively associated with seeing profoundness in bullshit statements and that those who have favorable views of Donald Trump, Ted Cruz, and Marco Rubio are more likely to judge bullshit statements as profound compared with individuals who have less favorable views of these candidates.

Fourth, the present research remains empirically silent regarding the explanatory variable of why conservatism is positively associated with seeing profoundness in bullshit statements. We base our study on the empirically supported assumption found in other research that conservatives (vs. liberals) are less likely to use a reflective and critical thinking mode [8,9], a mode that is necessary to detect the vacuity of pseudo-profound bullshit statements [1]. However, reflective and critical thinking is not measured in the present study. The assumption that reflective and critical thinking can explain the found effects needs to be tested in future research. Additionally, as bullshit receptivity is related to religiousness [1], and while it is likely that religiousness is positively related to favorable views of Cruz, it could be that religiousness might explain (part of) the found relations. Since religiousness is not assessed in the present study (which also holds true for other demographic variables such as social class, income, and education) there is room for future research to test variables that might explain the found relations.

Fifth, it is likely that conservatives are specifically receptive to pseudo-profound bullshit but not to others forms of bullshit. To detect pseudo-profound bullshit, a critical thinking mode is necessary, a mode that is less likely to be found in conservatives [5,8,9]. Another thinking mode may be required to detect other forms of bullshit, for instance, when people tell sincere exaggerations that are detectable without critical thinking [14]. As such, it seems possible that conservatism is not related to seeing any form of bullshit as profound.

Notably, it has recently been discussed whether the items of the Bullshit Receptivity scale actually reflect pseudo-profound bullshit [15,16]. Dalton [15] argues that some items (e.g., “Wholeness quiets infinite phenomena.”) might reflect meaningful, profound sentences from a transcendent Buddhist perspective, and thus do not represent pseudo-profound bullshit (or even true) statements in the eyes of some perceivers. Although this might well be the case, according to Pennycook and colleagues [1,16], bullshit is not defined by the perceiver but by the sender. Given that the bullshit statements used consist of randomly selected buzzwords, they fulfil the definition of a communicative expression that lacks plausibility and truth from the perspective of natural science. Thus, as Pennycook and colleagues ([16], p. 123) put it, “[b]ullshit that is viewed as profound is still bullshit.”

To conclude, the present work shows that political conservatism is positively related to seeing profoundness in pseudo-profound bullshit statements. It may be that this finding and the present research in general has an impact on some conservatives in that they might evaluate statements more critically. We invite individuals to start with the present contribution.

## Supporting Information

S1 File Data. (ZIP)
